# Estimation of physician supply by specialty and the distribution impact of increasing female physicians in Japan

**DOI:** 10.1186/1472-6963-9-180

**Published:** 2009-10-07

**Authors:** Soichi Koike, Shinya Matsumoto, Tomoko Kodama, Hiroo Ide, Hideo Yasunaga, Tomoaki Imamura

**Affiliations:** 1 Department of Planning, Information and Management, University of Tokyo Hospital, Tokyo, Japan; 2 Department of Policy Sciences, National Institute of Public Health, Saitama, Japan; 3 Department of Health Management and Policy, Graduate School of Medicine, University of Tokyo, Tokyo, Japan; 4 Department of Public Health, Health Management and Policy, Nara Medical University, Nara, Japan

## Abstract

**Background:**

Japan has experienced two large changes which affect the supply and distribution of physicians. They are increases in medical school enrollment capacity and in the proportion of female physicians. The purpose of this study is to estimate the future supply of physicians by specialty and to predict the associated impact of increased female physicians, as well as to discuss the possible policy implications.

**Methods:**

Based on data from the 2004 and 2006 National Survey of Physicians, Dentists and Pharmacists, we estimated the future supply of physicians by specialty, using multistate life tables. Based on possible scenarios of the future increase in female physicians, we also estimated the supply of physicians by specialty.

**Results:**

Even if Japan's current medical school enrollment capacity is maintained in subsequent years, the number of physicians per 1000 population is expected to increase from 2.2 in 2006 to 3.2 in 2036, which is a 46% increase from the current level. The numbers of obstetrician/gynecologists (OB/GYNs) and surgeons are expected to temporarily decline from their current level, whereas the number of OB/GYNs per 1000 births will still increase because of the declining number of births. The number of surgeons per 1000 population, even with the decreasing population, will decline temporarily over the next few years. If the percentage of female physicians continues to increase, the overall number of physicians will not be significantly affected, but in specialties with current very low female physician participation rates, such as surgery, the total number of physicians is expected to decline significantly.

**Conclusion:**

At the current medical school enrollment capacity, the number of physicians per population is expected to continue to increase because of the skewed age distribution of physicians and the declining population in Japan. However, with changes in young physicians' choices of medical specialties and as the percentage of female physicians increases, patterns of physician supply will vary between specialties. Specialties less often chosen by young physicians and where males have dominated will face a decline in physician supply. These results highlight the necessity for developing a work environment that attracts female physicians to these types of specialties. This will also lead to improved gender equality in the workforce and more effective use of human resources.

## Background

Since training new physicians is a long process and requires significant resources from both taxpayers and physicians themselves, one of the most common health policy issues faced throughout the world is matching physician supply with the needs and preferences of the population. Estimating the future physician supply provides an evidence basis for appropriate health policy. For this purpose, transitions in a society's healthcare policies and transitions in physicians' workplace preferences, as well as the demographics of the physicians themselves, should be taken into account. Therefore, a country analysis of these factors can provide useful information as a discussion base for health policymakers worldwide.

In Japan, there have been three significant changes affecting physician supply and distribution in recent years. They are: 1) a change in the post-graduate clinical training system; 2) an increase in medical school enrollment capacity; and 3) an increase in the percentage of female physicians.

The first change came with the introduction of the new post-graduate clinical training system in 2004. The new system shifted new graduates from academic hospitals to non-academic hospitals. In 2001, before the introduction of the new system, 70% of clinical trainees were receiving their clinical training at academic hospitals. About 40% of clinical trainees were receiving their training in a particular specialized field at an institution affiliated with their medical school. The distribution of physicians changed significantly in 2004 when the new system was introduced. Under this system, a matching system was introduced at the national level to match applicants' priority lists for training facility choices and the training facilities' lists of preferred candidate and available resident positions. As more non-academic hospitals offered training positions, the number of clinical trainees at academic hospitals dropped below 50%.

The second change was the increase in medical school enrollment capacity. The number of physicians per 1000 population was 1.1 in 1970, which was low compared with other developed countries. In 1970, the country's total first-year medical school capacity was 4,380. Then the government set the target number of physicians at 1.5 per 1000 population. This led to the establishment of a number of new medical schools, and in 1985 the enrollment capacity reached a peak of 8,340. After the target physician number was reached, the "Committee for the Future Supply-Demand of Physicians" recommended in 1986 that the number of new physicians be reduced by at least 10%, and subsequently the number of new physicians began to decline [1]. In 2008, the Committee for Studying the Realization of the Desired Medical Care System released their Interim Report. The committee recommended an increase in the number of new physicians in Japan. The report emphasized to the government that "the number of new physicians produced in the next fiscal year (2009) must surpass the past highest medical school enrollment capacity (8,360) and ... should be increased by about 50% in the future" [2]. Subsequently, in the 2009 fiscal year, the medical school enrollment capacity was set to 8,486, which is the highest level ever.

The third significant change was the increase in the proportion and the number of female physicians. The percentage of female physicians in the workforce rose from 9.8% in 1975 to 17.2% in 2008. Female medical students now account for over 30% of the total number of physicians who pass the national examination. This indicates that a proper discussion of physician supply and the gender composition of medical specialties needs to focus more on female physicians' preferences and career patterns.

Since the introduction of the new clinical training system in 2004, various studies and reviews have been published on the status of physicians who have recently completed their initial clinical training [3,4]. A provisional estimation of the supply and demand of physicians has recently been presented at a national review committee meeting [5]. Regarding the impact of the increased number of female physicians, the results of an analysis on the recent status of the number of female physicians and the employment rate of female physicians are available [6]. However, a detailed analysis and comparison by gender and types of medical specialties has not yet been carried out. The purpose of this study is to estimate the future supply of physicians and their distribution among specialties, by focusing on the increase in medical student enrollment capacity and the increase in female physicians, and to discuss the related policy implications. Our results can provide a wider research base for policy discussion in other countries.

## Methods

For this study, we used data from the 1996 through 2006 data of the National Survey of Physicians, Dentists and Pharmacists, supplied by the Ministry of Health, Labour and Welfare, Japan. The National Physicians' Law requires all physicians to report their status every two years. The data included the physician registration number, gender, age, workplace municipality code, and self-designated specialty. The survey asks for the physicians' self-designated specialty and not their board/certified specialty. This point needs to be considered when interpreting our data. The relationship between a physician's self-designated specialty and board certification varies, and they are not necessarily the same. For example, whereas the number of self-designated pediatricians (14,700 as of the end of 2006) is similar to the number of board-certified pediatricians (12,354 as of March 2008), the number of self-designated rheumatologists (760 as of the end of 2006) is far less than the number of board-certified specialists registered with the Japan College of Rheumatology (3,701 as of March 2008).

The data for 270,353 physicians from the 2004 survey and 277,927 from the 2006 survey were used to carry out the future estimation of physicians. The data from 1996 to 2006 were used to analyze trends in physicians' specialty choices. The researchers were not able to identify individual physicians from the data provided. To carry out the estimation of the future number of physicians by specialty, a multistate life table was used. A conventional life table only indicates "dead" or "alive," while a multistate life table deals with broader status categories [7,8]. First, we identified seven medical specialty status categories (six different specialties groups plus a "not reported" status). Then, the physician distribution among the specialties according to years of work experience was determined using the survey data from 2004 and 2006. By assuming that the probability of status category changes in a given year of experience is constant, the distribution of physicians for the year n+2 can be obtained by multiplying the number of physicians in year n by the probability of change of specialty status category (practicing, non-practicing, or change of specialties if applicable) in a given year of experience. By continuing this process, the total number of physicians by specialty was forecast for each year up to the year 2036.

In estimating the number of physicians by specialty, possible scenarios were proposed with regard to the numbers of new physicians and female physicians. The first simulation was to estimate the number of physicians in each medical specialty on the assumption that the future yearly number of new physicians stays at the 2009 level (school enrollment capacity = 8,486). The total number of internists and surgeons, and the number of psychiatrists per 1000 population, obstetrician/gynecologists (OB/GYNs) per 1000 births, and pediatricians per 1000 population under 15 years of age were calculated.

The second simulation was to estimate the number of physicians in each medical specialty using each of the three following assumptions regarding the proportion of female physicians: 1) the future percentage of new female physicians remains at the 2006 survey level (32.8%); 2) it increases to 40% in 10 years; and 3) it increases to 50% in 10 years. Then the number of physicians by specialty was estimated and the total number of internists and surgeons, psychiatrists per 1000 population, OB/GYNs per 1000 births, and pediatricians per 1000 population under 15 years of age were calculated.

The multistate life table calculation program "MSLT" [9] was used to obtain the multistate life tables and the future estimation. The number of physicians per 1000 population, the number of pediatricians per 1000 population under 15 years of age, and the number of OB/GYNs per 1000 births were calculated based on the report "Population Projections for Japan: 2006-2055 (medium-variant fertility [with medium-variant mortality])" from the National Institute of Population and Social Security Research [10].

## Results

### (1) Characteristics of physicians in the 2004 and 2006 National Survey of Physicians, Dentists and Pharmacists

Table 1 presents the characteristics of the physicians from the 2004 and 2006 surveys. The percentage of female physicians increased from 16.5% in 2004 to 17.2% in 2006, and these increases were observed in all age groups except for the "over 70" category.

**Table 1:** Characteristics of physicians in the 2004 and 2006 surveys

	**2004 Survey**			**2006 Survey**		
	
	**Male**	**Female**	**Total**	**Male**	**Female**	**Total**
Total Reported	225,731	44,622	270,353	230,043	47,884	277,927
Average Age	48.91	41.98	47.77	49.29	42.06	48.05
Age Group						
Under 29	17,084	9,333	26,417	16,922	9,428	26,350
30-39	51,448	14,922	66,370	50,656	16,401	67,057
40-49	61,610	9,679	71,289	60,383	10,409	70,792
50-59	44,074	5,012	49,086	50,703	5,903	56,606
60-69	22,806	2,026	24,832	22,692	2,238	24,930
Over 70	28,709	3,650	32,359	28,687	3,505	32,192
						
Place of Work						
Academic Hospitals	38,139	9,327	47,466	38,553	10,100	48,653
Non-Academic Hospitals	100,882	19,370	120,252	102,782	20,857	123,639
Clinics	79,071	13,911	92,982	80,459	14,754	95,213
Other Facilities	7,639	2,014	9,653	8,249	2,173	10,422
						
Specialties						
Internal Madicine	87,301	14,438	101,739	85,673	14,169	99,842
Surgery	52,168	2,658	54,826	50,866	2,623	53,489
Pediatrics	10,105	4,572	14,677	10,124	4,576	14,700
Obstetrics and Gynecology	9,461	2,695	12,156	9,022	2,761	11,783
Phychiatrics	10,285	2,315	12,600	10,438	2,391	12,829
Other Specialties/Others	56,411	17,944	74,355	63,920	21,364	85,284

With regard to the distribution of male and female physicians by specialty, the percentage of male surgeons (around 95%) was much higher than that of their female counterparts (around 5%). On the other hand, the percentage of female pediatricians was relatively high (compared with the overall percentages of men and women physicians), with female pediatricians accounting for 31% of that specialty. Although the total number of physicians reported in 2006 (277,927) was slightly higher than that in 2004 (270,353), the number of physicians specializing in internal medicine, surgery, and pediatrics declined in 2006.

### (2) Estimated number of physicians up to 2036

**Figure 1:**
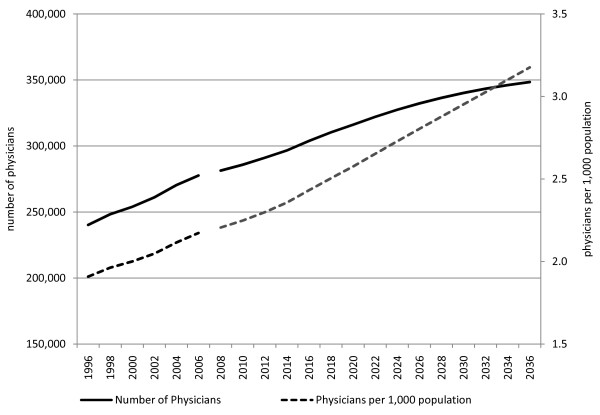
**Number of physicians and physicians per 1000 population (1996--2006: trends; 2008--2036: estimates)**. The trends and estimated total number of physicians per 1000 population and actual number of physicians are presented. The number of physicians per 1000 population is estimated to reach 3.0 in 2032 and 3.2 in 2036, which is about a 1.5-fold increase from the 2006 level. The actual number of physicians will continue to increase, reaching 303,800 by 2016, then 348,400 by 2036. The rate of increase will peak between 2014 and 2016 and then will begin to slow down gradually. The increase rate will stay constant through the 2030s to reach a saturated level.

Our estimation showed that, even with the medical school enrollment capacity set at the current level, the number of physicians per 1000 population will continue to increase from 2.2 (total number: 277,900) in 2006, reaching 3.0 (total number: 343,400) in 2032 and later 3.2 (total number: 348,400) in 2036, which is a 46% increase from the 2006 level. The actual number of physicians will not show a constant increase; the increase rate will peak between 2014 and 2016 and then will begin to slow down gradually. The increase in the number of physicians will stay constant at around 1000 annually in the 2030s. Meanwhile, the number of physicians per 1000 population will see a constant increase (See Figure 1).

**Figure 2:**
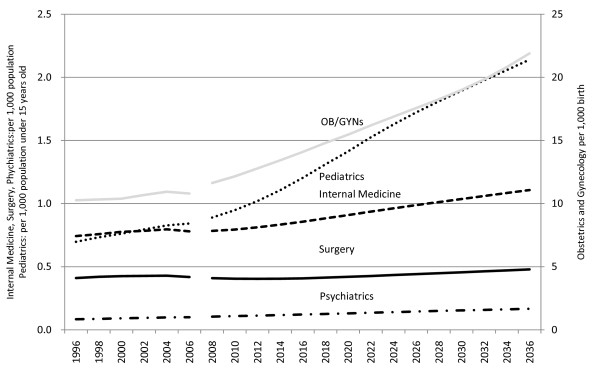
**Trends and estimates of physicians in different specialties (1996--2006: trends; 2008--2036: estimates)**. The trends and estimated number of physicians per target population are presented. The number of internists, surgeons, and psychiatrists per 1000 population (left y-axis), pediatricians per 1000 population under 15 years of age (left y-axis), and OB/GYNs per 1000 births (right y-axis) are shown.

With regard to the future estimate of physicians by specialty per target population, all but the number of surgeons per 1000 population will increase (See Figure 2.). As for the actual number of physicians by specialty, the number of OB/GYNs is expected to decline temporarily from 11,800 in 2006 to 11,400 in 2010. This number is later expected to increase slightly, but to remain below the 2006 level until 2018, after which it would again increase to 13,900 by 2036. The number of surgeons is expected to decrease from 53,500 in 2006 to 51,000 in 2016. The number is then expected to increase slightly, but to remain below the 2006 level at 52,600 by 2036.

### (3) The impact of a further increase in the percentage of female physicians

By 2036, the combined total of male and female physicians per 1000 population is expected to reach 3.2 if the number of new physicians remains at the current level, 3.2 if the number of new female physicians increases to 40%, or 3.1 if the number of new female physicians increases to 50%.

**Figure 3:**
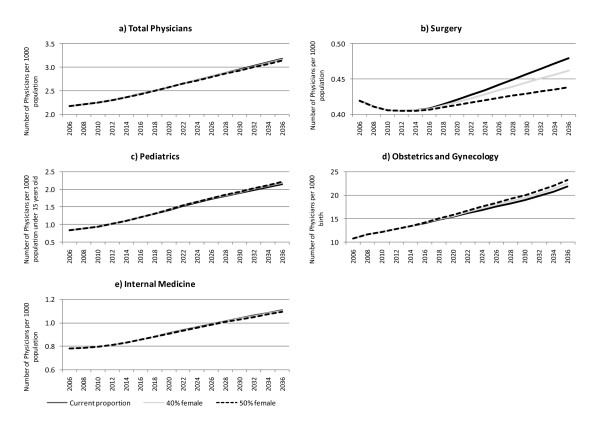
**Increase in female physicians and the effect on the number of physicians**. Estimates are presented of the number of physicians per target population by selected specialty using three scenarios regarding female physicians: 1) the future percentage of new female physicians remains at the current level; 2) it increases to 40% in 10 years; and 3) it increases to 50% in 10 years. a) The total number of physicians per 1000 population by 2036 is expected to reach around 3.2 for the current level, 3.2 for the 40% female scenario, and 3.14 in the 50% female scenario. The number of b) internists and c) surgeons and per 1000 population are expected to decrease as the percentage of female physicians increases in all three scenarios. The numbers of d) pediatricians per 1000 population under 15 years of age and e) OB/GYNs per 1000 births are expected to increase as the percentage of female physician increases.

With regard to the number of physicians by specialty per target population, the numbers of pediatricians and OB/GYNs are expected to increase as the percentage of female physicians increases. Meanwhile, the number of internists is expected to decrease as the percentage of female physicians increases. If female physicians account for 40% of the workforce, the number of surgeons per 1000 population is expected to decrease from 0.419 (total number: 53,500) in 2006 to 0.408 (total number: 50,900) in 2012, then increase slightly to 0.462 (total number: 52,600) by 2036. If female physicians account for 50% of the workforce, the number of surgeons per 1000 population is estimated to decrease to 0.405 (total number: 50,900) in 2012, then increase slightly to 0.438 (total number: 48,100) by 2036. (See Figure 3).

## Discussion

### Estimated number of physicians up to 2036

The physicians who are now reaching retirement age are those who received their medical licenses when the medical school enrollment capacity was still small, and thus retiring physicians are now far outnumbered by new physicians. This is the main reason why the number of physicians is expected to continue to increase in the future even if the enrollment capacity stays at the current level. The declining Japanese population, which is estimated to contract from 127.8 million in 2005 down to 119.3 million by 2015 and then to 109.7 million by 2036, will also accelerate the rate of increase in the number of physicians per population.

Currently, the number of physicians per 1000 population in Japan ranks near the bottom among the OECD member countries [11]. There have been intensifying discussions on the shortage of physicians and this has led to the development of a policy measure to increase the number of new physicians in the country [12]. However, a sufficient supply of physicians is a prerequisite but not the only necessary factor in order to achieve cost-effective healthcare [13], and since it is difficult to project an adequate ratio of physicians to patients [14], the supply and demand of physicians should not be contemplated solely based on the number of physicians.

The unequal distribution of physicians by specialty is now an urgent policy issue. While the number of OB/GYNs per 1000 births will continue to increase, mainly as the number of births decreases, obstetrics is one particular specialty field that draws public attention and is expected to be the target of urgent public health policy action. The reason for this is the current lack of multiple-obstetrician on-duty teams and the lack of regional 24-hour inpatient obstetrics facilities for high-risk patients. These factors are contributing to the relatively high mortality rate among pregnant women in Japan [15]. There has been a decrease in the hiring of newly registered OB/GYNs, and many of those originally practicing in obstetrics and gynecology later leave the specialty to practice in internal medicine [16].

Changing physicians' medical specialties to balance the workforce would take a long time and would not be the best use of limited health care resources, while employing non-Japanese physicians is difficult due to the language barrier. As such, it is important in the medium to long term in Japan to provide incentives for new medical school graduates and those who have recently finished their post-graduate clinical training to make appropriate specialty choices that are coordinated in a balanced manner. In the short term, however, discussions on coordinating the sharing of responsibilities between physicians and their medical coworkers and between different types of medical facilities are necessary.

### The impact of a further increase in the percentage of female physicians

The overall number of physicians will decrease slightly but not change significantly if the percentage of female physicians further increases. This is mainly because the workforce participation rate for female physicians is lower than that of their male equivalents, even though the life expectancy of females is longer than that of males. However, in medical specialties where male and female physicians are unevenly distributed, the impact of a further increase in the percentage of female physicians on the overall supply of physicians will be more significant.

The growing number of female physicians leads to a relative decrease in the proportion of their male counterparts, leading to an overall decline in the total number of physicians in medical specialties where the percentage of female physicians is significantly lower. The impact is particularly significant in the field of surgery; in addition to the percentage of female physicians being extremely small (male physicians account for 95% of all surgeons), the proportion of male physicians choosing surgery as a specialty is also decreasing. With these two factors combined, the total number of surgeons will decrease sharply.

The increase in the number of female physicians is not a phenomenon exclusive to Japan and so the experience and lessons gained from this study can also be applied to other countries. In the United States, it was pointed out over 20 years ago that an increase in the number of female physicians could have a significant impact on the future supply and demand of physicians, as female physicians tend to choose medical specialties different from those chosen by their male counterparts, and many female physicians choose to work only in urban areas [17]. Studies conducted in Nordic countries also suggested that the rate of increase in the number of female surgeons was slow, though the range of medical specialties female physicians tended to choose was growing and the percentage of female surgeons was gradually increasing [18] as the number of female physicians increased. In the United States, the impact of the increase in the number of female physicians on work-family balance and employment status was studied [19]. It was found that female physicians tend to work fewer hours. To compensate, more physicians should be trained and new programs to support medical care should be implemented, particularly in rural areas.

It is necessary that adequate actions be taken for each type of medical specialty to effectively rectify the lack of physicians, especially in fields where male and female physicians are distributed unevenly.

In the current situation, policies designed to influence specialty distribution are rather incentive-based, rather than controlling the supply by limiting the physician number in certain specialties. Japanese Medical Law does not limit medical practices to within their physicians' specialty areas, and no nationally-agreed upper limit has been placed on the number of specialty certification numbers. The Japanese specialty system was developed based on each academic society rather independently. Physicians who have completed their two-year initial post-graduate clinical training and those who seek to become board-certified specialists undergo training in designated hospitals and then take the board examination. Efforts and discussion are still continuing to better coordinate and to establish common ground among different specialties' certification system under the auspices of the Japanese Board of Medical Specialties, which was established in 2002.

With regard to responding to the increase in female physicians, identifying female role models to inspire new female medical students to pursue particular specialties is one possible approach to guide female physicians' career choices. For example, the more female surgeon role models there are, the more female medical students will become interested in surgical practice [20]. Meanwhile, the medical profession has realized that long working hours and unstructured training do not tend to ensure a well-equipped and well-balanced workforce [21]. Improving the work environment to make it more favorable to women could lead to benefits for the whole medical care team, both males and females, and eventually to the establishment of a gender-balanced workplace.

### Limitations of this study and areas for future research

Discussed below are some limitations to this study as well as areas for further research, namely: 1) we assumed that the characteristics and status of physicians in the 2004-2006 period will continue into the future; 2) we calculated the number of physicians as a head count instead of the full-time equivalent (FTE); and 3) primary care and the role of different specialties and gender require further study.

First, our estimation method was based on the assumption that the trends in physicians' choices of medical specialties in 2004-2006 will continue unchanged. Therefore, the accuracy of our medium- and long-term estimates could be affected by the possibility that present career choice patterns may no longer apply as the systems for educating and training physicians undergo significant changes. This assumption also applies for the women's preferences for specialties, in that today's preferences were assumed to continue into the future and stay constant. However, as more female physicians enter medical professions and specialties, their preferences may change.

Second, this study estimated only the number of physicians, not the FTE number. The working patterns of physicians are diverse, due to the introduction of shorter shifts and shift-work systems, but this study could not differentiate the number of physicians in full time and part-time positions. Part-time shift is seen among pediatricians in the United States, where it has been revealed that many female pediatricians tend to seek and obtain part-time work [22]. However, we believe the basic data we were able to obtain through this study is as accurate as possible, since data on the actual working status of physicians is not available. We feel the results of this study can provide useful information for policy debates if the assumptions we set are taken into account.

Third, primary care and the role female physicians should be the focus of further research. The World Health Report 2008 revisited the importance of primary health care and the operation of national health systems. The report pointed out that the world's health systems face three worrisome trends: 1) a disproportionate focus on a narrow range of available specialized treatment and care; 2) a focus on short-term results with fragmented service delivery; and 3) a hands-off or laissez-faire approach to governance [23]. This report provides a useful framework for tackling the related issues in Japan. Possible policy options to deal with inequalities in the distribution of physicians among medical specialties must be considered. There should be a shift away from a narrow focus on specialized care towards making primary care more predominant, so as to achieve comprehensive service delivery. An appropriate level of policy intervention needs to be introduced with close collaboration of the government, the medical community, and civil society. All of these discussions need to be based on available evidence in order to attain the potential positive outcomes.

In many countries, female medical students are more likely to become primary care clinicians [24], and this increase in female physicians will positively affect the primary care field. This phenomenon is not the same in Japan. There is no standard specialty term or professional organization for "general internists" or "family physicians" in Japan [25]. Meanwhile, physicians in office-based practices often do not limit their practice to the specialty in which they were trained [26].

In addition, research on the role of gender in choosing different specialties is not enough to encourage a greater primary care focus. For example, it is considered that many physicians who registered as general internal medicine and pediatrics specialists later become involved in primary care. Thus, it may be deduced that increasing the number of female physicians will result in an increase in general internal medicine practitioners and pediatricians and thus ultimately more physicians practicing primary care. However, in Japan, the gender balance in specialty choices is disproportionate and has a multifaceted influence on primary care physician numbers. Japan's 2006 National Physicians Survey shows that 14.6% of male physicians and 10.1% of female physicians chose pediatrics, and 27.5% of males and 23.3% of females chose general internal medicine, while 2.2% of males and 6.8% of females chose dermatology, and 3.6% of males and 10.1% of females chose ophthalmology. So, if the current preference trends in choice of specialty continue, increasing the female physician proportion does not directly lead to an increase in physicians who provide a primary care oriented practice. Thus, further research on primary care is needed, and the establishment of primary care as a unique medical discipline in Japan is also necessary.

This study presents useful estimates of the supply of physicians by medical specialty, and predictions of the possible impacts of the increased numbers of new physicians and female physicians. With an understanding of the limitations and assumptions of this estimation model, our results may have significant policy implications for other countries faced with similar issues.

## Conclusion

This study estimated the future supply of physicians and their distribution among specialties, by focusing on the increase in medical student enrollment capacity and the increase in female physicians, and discussed the policy implications based on these estimates.

The number of physicians per 1000 population is expected to increase in the future due to the skewed age distribution of physicians and the continuing decline in the Japanese population, even if the medical school enrollment capacity remains unchanged from the 2009 level. The number of surgeons and obstetricians could decrease further even if the total number of physicians increases, since the physicians in these fields have recently shown increased intentions to leave their specialty practice, while the number of new physicians who choose these specialties is decreasing.

This study also suggests that the increase in the number of female physicians could impact certain specialties where the percentage of female physicians was originally small, and which both male and female new physicians tend to refrain from choosing. Thus, it is necessary to develop a working environment in these specialties that is favorable to female physicians in terms of gender equality and the effective use of human resources.

## Competing interests

The authors declare that they have no competing interests.

## Authors' contributions

**SK **conceived of the study and participated in the study design, literature review, data analysis, and manuscript drafting. **SM **participated in the simulation, data analysis, and manuscript drafting.**HI **participated in the data cleaning and manuscript drafting. **HY**, **TK**, and **TI **participated in the manuscript drafting. All authors discussed the results, commented on the manuscript, and gave their final approval.

## Pre-publication history

The pre-publication history for this paper can be accessed here:


